# Effects of Moisture on NH_3 _Capture Using Activated Carbon and Acidic Porous Polymer Modified by Impregnation with H_3_PO_4_: Sorbent Material Characterized by Synchrotron XRPD and FT-IR

**DOI:** 10.3390/ma15030784

**Published:** 2022-01-20

**Authors:** Chu-Chin Hsieh, Jyong-Sian Tsai, Jen-Ray Chang

**Affiliations:** 1 Department of Safety, Health and Environmental Engineering, National Yunlin University Science and Technology, Douliu 640301, Taiwan; hsiehcc@yuntech.edu.tw; 2 Department of Chemical Engineering, National Chung Cheng University, Chia-Yi 621301, Taiwan; admjstsai@ccu.edu.tw

**Keywords:** NH_3_ capture, activated-carbon-supported H_3_PO_4_ sorbents, porous acidic-polymer-supported H_3_PO_4_ sorbents, synchrotron XRPD, FT-IR, breakthrough curve

## Abstract

The performances of reactive adsorbents, H_3_PO_4_/C (activated carbon) and H_3_PO_4_/A (Amberlyst 35), in removing NH_3_ from a waste-gas stream were investigated using a breakthrough column. Accelerated aging tests investigated the effects of the water content on the performance of the adsorbents. Results of breakthrough tests show that the adsorption capacity greatly decreased with the drying time of H_3_PO_4_/C preparation. Synchrotron XRPD indicated increased amorphous phosphorus species formation with drying time. Nitrogen adsorption-desorption isotherms results further suggested that the evaporation of water accommodated in macropores decreases adsorption capacity besides the formation of the amorphous species. Introducing water moisture to the NH_3_ stream increases the adsorption capacity concomitant with the conversion of some NH_4_H_2_PO_4_ to (NH_4_)_2_HPO_4_. Due to the larger pore of cylindrical type and more hydrophilic for acidic porous polymer support, as opposed to slit-type for the activated carbon, the adsorption capacity of H_3_PO_4_/A is about 3.4 times that of H_3_PO_4_/C. XRPD results suggested that NH_3_ reacts with aqueous H_3_PO_4_ to form NH_4_H_2_PO_4,_ and no significant macropore-water evaporation was observed when acidic porous polymer support was used, as evidenced by N_2_ isotherms characterizing used H_3_PO_4_/A.

## 1. Introduction

Ammonia is a hazardous gas. Besides its high flammability, ammonia at 50 ppm can irritate the eyes, throat, and nose and the detection threshold for humans is between 5 and 18 ppm [1,2]. Atmospheric NH_3_ produced by the biogenic decomposition of organic material, biomass burning, and fertilizer production has been the main contributor to ammonia emission [3,4,5]. Ammonia emission from agricultural and nonagricultural usually is less than 100 ppm [1,2]. The others are contributed from the use and the production of synthetic nitrogen fertilizer, waste treatment, and fuel combustion [5,6]. Many techniques have been developed to abate NH_3_ emission from livestock farming, such as absorption in solution by acid scrubber, biodegradation in a biotrickling filter, and adsorption by porous solids. Among these techniques, NH_3_ removal efficiency for biotrickling filters is relatively lower at high NH_3_ concentrations because of the inhibition of nitrifying bacteria [7,8]. Wet acid scrubbing is an effective and inexpensive process, but the utilization of acid scrubber has to suffer scaling inside the scrubber and corrosion of the equipment [9,10].

Adsorption of NH_3_ on porous solids, such as alumina or alumina-bound-zeolite pellets, seems a promising approach due to its simplicity and economy in configuration and operation [11,12]. Zeolites, silica-alumina, and alumina can be excellent adsorbents for ammonia removal when they are used in treating dry waste gas. However, with the presence of water moisture, the gradual reaction of NH_4_OH with alumina erodes the solid pellets, resulting in increased pressure drop of the adsorption bed, which increases the difficulty in operation. Moreover, the great quantity of used adsorbent has to be disposed of by using costly landfills. The erosion problem under wet conditions may be eased by using activated carbon-supported mineral acid [13,14,15,16], and mesoporous silica-supported N-halamines [17].

Compared with zeolite, silica-alumina, and alumina pellets, mesoporous silica has higher hydrothermal stability [17]. Granular activated carbon, however, not only exhibits high surface area but has high acid and alkaline resistance, allowing the impregnation of acid or base solutions without damaging supports [10,13]. The instantaneous reaction of the acid solution and N-halamines impregnants with NH_3_ significantly increases the NH_3_ removal capacity [17]. 

Activated carbon impregnated with H_2_SO_4_ (H_2_SO_4_/C) has been prepared for the removal of NH_3_ in dry [15] and wet waste gas streams [10,18]. Impregnants, H_2_SO_4_, on the sorbent H_2_SO_4_/C, react with NH_3_ in the waste gas stream to form stable (NH_4_)_2_SO_4_ salts. The NH_3_ removal capacity of H_2_SO_4_/C is more than that of the activated-carbon adsorbents. In addition, the used H_2_SO_4_/C can be rejuvenated by washing (NH_4_)_2_SO_4_ salts with low-pressure steam and then re-soaking H_2_SO_4_ solutions [10]. However, H_2_SO_4_ is a highly corrosive strong mineral acid. In re-soaking H_2_SO_4_ on the active carbon, the adsorption column is gradually corroded by the stagnant H_2_SO_4_ solution. Moreover, H_2_SO_4_ is a strong oxidizer and dehydrating agent. Because of its strong oxidizing power, H_2_SO_4_ acid may react violently with organic compounds formed from biodegradation of livestock waste, resulting in gas evolution and potential pressure buildup in the adsorption column.

The structure of phosphoric acid, H_3_PO_4_, is similar to that of H_2_SO_4_, whereas H_3_PO_4_ tends to be much less acidic and corrosive and has less oxidizing power. The potential hazardous of H_3_PO_4 _is much less weak than that of H_2_SO_4_. Hence, activated carbon impregnated with H_3_PO_4_ (H_3_PO_4_/C) samples were prepared in this study to remedy the drawbacks of H_2_SO_4_/C. Like H_2_SO_4_/C, the spent H_3_PO_4_/C can also be regenerated by flowing low-pressure steam through the adsorbent bed and the regeneration byproduct (NH_4_)_x_H_3-x_PO_4_ [19] can be used as agricultural fertilizer.

Although activated carbon exhibits excellent adsorption properties due to its high surface area, the pores of impregnated activated carbon (IAC) may easily be blocked by salts formed from the reaction of a noxious-gas adsorbate with the impregnated H_3_PO_4_ due to quick evaporation of water [10]. This study also used functionalized porous polymer (FPP) for H_3_PO_4_ impregnation besides activated carbon to decrease the water evaporation rate. The motivation for using FPP is that the pore structure and surface functional group of FPP can be tuned by using monomers of various sizes, shapes and by linking functional moieties to them [20,21,22]. Ambelyst 35 was chosen in this study because the average pore of this material is about 300 Å, and the resin abounds with sulfonic acid sites, which minimize the pore plugging in the NH_3_ removal process and facilitate the adsorption of NH_3_ on the surface due to its high water-retaining capacity.

## 2. Experimental 

### 2.1. Sample Preparation

The carbon-supported reactive adsorbent, H_3_PO_4_/C (noted as PC), was prepared by impregnating activated carbon (GAC 830) with a 7 M H_3_PO_4_ solution. The same procedure was used to prepare H_3_PO_4_/A adsorbent with Amberlyst 35 support (noted as PA). The activated carbon (GAC 830 of particle size, 2 to 5 mm; surface area, 1050 m^2^/g; pore volume, 0.85 mL/g; iodine No., 75 mg/g minimum and apparent density, 0.54 g/mL) was purchased from Norit Americas Inc., Atlanta, GA, USA. The acidic porous polymer (Amberlyst 35 of particle size, 0.30 to 0.85 mm; surface area, 50 m^2^/g; pore volume, 0.35 mL/g; and apparent density, 0.56 g/mL) support was purchased from Rohm and Haas company. In preparing the reactive adsorbents, the activated carbon or the acidic porous polymer were dried at 120 °C by passing through air to remove physically adsorbed water. The dried supports of 120 g were brought into contact with 180 g phosphoric acid solution. Upon soaking for 30 min, the reactor for the preparation was purged with N_2_ for 60 min. The resulting material was noted as H_3_PO_4_/C and H_3_PO_4_/A, respectively, for GAC830 and Amberlyst 35 supports. H_3_PO_4_/C and H_3_PO_4_/A samples were then exposed to dry air for 24 h. These samples were noted as PC0 and PA0. In order to study the water effects, PC0 was dried in an air environment at 120 °C for 4, 8, and 24 h. (the samples are noted as PC4, PC8, and PC24), while PA0 was dried for 14 h (PA14) for comparison. 

### 2.2. Breakthrough Curve Tests

The adsorption capacity under dynamic conditions for ammonia removal was carried out in a stainless-steel fixed bed adsorption-regeneration column of 2.2 inside diameter and 45 cm length. The column was packed with 10.0 g of H_3_PO_4_/C or H_3_PO_4_/A with inert ceramic (sphere of about 2.0–5.0 mm diameter) in a ratio of 1:10 by volume. The diluent was used to minimize channeling effects. Typically, the performance tests used an ammonia concentration of 8.4 mole % NH_3_ in the air stream (0.76 L/min). The effluent gas was passed through an HCl solution scrubber to remove the unremoved NH_3_. Mass flow controllers were used for controlling the NH_3_ and total flow rate. The NH_3_ absorbed in the scrubber was monitored by a pH meter, and a breakthrough curve was plotted by plotting the pH value with time. The total amount of NH_3_ removal was calculated from the breakthrough curve. For studying the performance of PC0 for moisture-containing waste gas, noted as PC0 (wet), the airstream was humidified by passing through a water reservoir before mixing with NH_3_. 

### 2.3. Synchrotron XRPD

X-ray powder diffraction (XRPD) was performed at the BL01C2 beamline of the National Synchrotron Radiation Research Center (NSRRC) (Hsinchu, Taiwan). The X-ray source of BL01C2 is a 5.0 T superconducting wavelength shift magnet which provided 8 to 33 keV X-rays. Wavelength λ = 0.08266 nm (15.0 keV) was chosen for the XRPD measurements. After a pre-focus mirror, a Si (111) double monochromator was used to select monochromator beams. Diffraction angle was calibrated with silver behenate and Si powders (NBS640b) standards according to their Bragg positions.

The samples were sealed in a glass capillary. The sample was fast spinning during the data collection to increase random orientations. Two-dimensional powder diffraction patterns were recorded by a Mar345 imaging plate and converted to a one-dimensional profile using the Fit2D V12.012 program (European Synchrotron Radiation Facility, Grenoble, France) [23]. Crystal structure parameters were refined with the Rietveld method [24] using the graphical interface package EXPGUI for GSAS program (Los Alamos, NM, USA) [25]. The calculated diffraction profiles were refined based on Pseudo–Voigt (Gaussian plus Lorentzian) profile function, and the broad background was fitted with a 22-parameter shifted-Chebyschev polynomial function. Since the occupancy and thermal parameters are highly correlated, the parameters of these two factors were refined alternatively with positional parameters. 

### 2.4. FT-IR Spectroscopy

Diffuse reflectance infrared Fourier transform spectra (DRIFT) of PC0 and PA0 samples were recorded with Shimadzu FT-IR, Prestige-21, with a deuterated triglycine sulfate (DTGS) detector. The spectra have a resolution of 2 cm^−1^. Before measurement, the samples were pulverized to powder. After taking the background KBr IR spectra, the powder samples were diluted with KBr and loaded into an in situ IR cell. NH_3_ in dry or wet air then was introduced into the IR cell and maintained for about 20 min for equilibrium. The cell was then purged with dry air for a few mins to remove free NH_3_, and IR spectra were recorded.

### 2.5. TGA Analysis

Thermogravimetric profile characterizing H_2_O containing in PC and PA were performed with a Q50 TGA (TA Instruments). The temperature control system includes a circular environment chamber, a sample pan furnace, and a purge gas supply system. In the testing, samples were heated up in the environmental chamber, which provides a stable temperature environment. Purging gas was provided from horizontal (60 mL/min air) and vertical (40 mL/min N_2_) directions. About 15 mg of gently ground samples were spread out in 100 μL Pt pans. The percent of weight loss as a function of temperature was determined at a heating rate of 5 °C/min.

## 3. Results and Discussion

### 3.1. Performance of H_3_PO_4_/C and H_3_PO_4_/A Characterized by Accelerated Aging Tests

To quickly evaluate the performance of H_3_PO_4_/C and H_3_PO_4_/A and obtain used sorbents for structure characterization, an accelerated aging test method has been developed and justified to save the test time [10,13].

**Figure 1:**
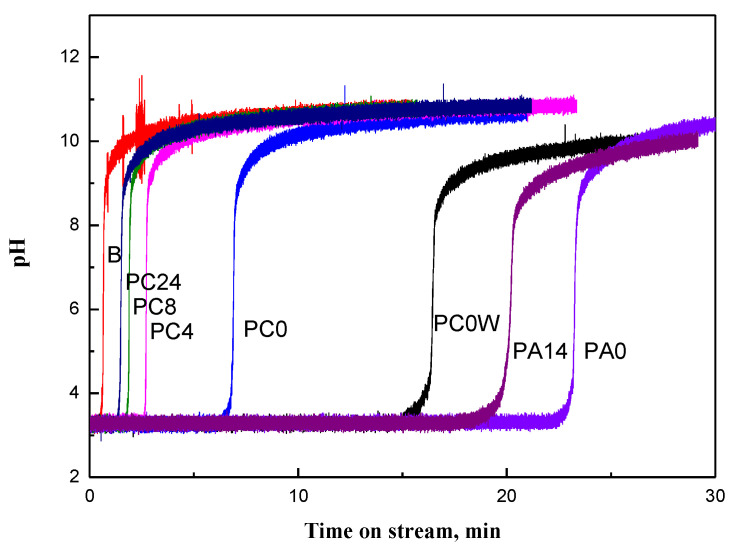
Breakthrough curve for different sorbents (from left to right: ceramic diluent, PC24, PC8, PC4, PC0, PC0_w_, PA14, PA0; subscript w: wet gas.

A blank test was conducted with an empty adsorption reactor packed with an inert ceramic diluent before the performance tests. As shown in Figure 1, the experimental results indicated that the experimental apparatus and diluent did not adsorb NH_3_ gas significantly, suggesting that the accelerating test would not be influenced by the adsorption and/or deposition of NH_3_ on the reactor and the diluent.

Our previous paper reported that the NH_3_ removal capacity for impregnated activated carbon (IAC), H_2_SO_4_/C, is about seven times that for HY zeolite (0.01 g NH_3_/g HY) [10]. Unlike adsorption of NH_3_ on activated carbon and zeolite, H_2_SO_4_/C removes NH_3_ in the waste gas by acid/base neutralization [10,15]. For H_3_PO_4_/C, NH_3_ in the waste gas can be removed by both neutralization and chemisorption. 

The NH_3_ removal process consists of three steps in series: 1, NH_3_ transport from the gas stream to the surface of H_3_PO_4_/C (PC) and was adsorbed on the external surface; 2, the adsorbed NH_3_ associated with water and diffuse or directly diffuse inside PC pores without water association; and 3, the reaction of NH_4_OH_(aq)_ or NH_3_ with H_3_PO_4(aq)_ to form mono-ammonium phosphate (MAP), di-ammonium hydrogen phosphate (DAP), and tri-ammonium phosphate (TAP), or the adsorption of NH_3_ inside pores of the sorbents.

The breakthrough curves characterizing the effects of drying time on PC’s performance for NH_3_ removal are shown in Figure 1. The time at which a significant concentration of NH_3_ breaks through the bed (breakpoint) is decreased with the drying time. Moreover, in contrast to the breakthrough profile of air-dried PC, a relatively gradual slope on the breakthrough curve has been observed for the PC sample without air drying (PC0). These results suggested that mass transfer resistance for air-dried samples, PC4, PC8, and PC24, is less than the resistance for PC0. The dissolution of NH_3_ in water could cause an increase in mass transfer resistance for PC0, whereas it increases NH_3_ removal capacity due to the reaction of NH_4_OH with H_3_PO_4_. 

For zeolite or active carbon adsorbents, moisture competes strongly with adsorbates, significantly reducing adsorption capacity [12]. However, comparing breakthrough profiles for PC with and without drying (Figure 1) suggests moisture is a necessary component in the process because, concomitant with the neutralization reaction, water in PC pores was removed by dry air. When water moisture is present in a waste gas stream, ammonia is dissolved in water before adsorption or reaction. Water is known to have a great affinity for adsorption in very small pores [26]; consequently, relatively more water is adsorbed on microporous pores of carbon than on mesoporous pores [26]. It has been reported that relatively more ammonia is adsorbed via dissolution in water present on the surface of the microporous carbon [27,28]. For waste gas containing sufficient moisture, such as effluent gas from the biological degradation of animal urine, the water content in PC is in equilibrium with gas humidity. Comparing the breakthrough profiles between PC0 and PC0(wet) demonstrates the advantage of water in the gas stream; the NH_3_ removal capacity for PC0(wet) is about 2.3 times that for PC0 (Figure 1).

H_3_PO_4_/A (PA) is more suitable for treating non-biological degradation ammonia emissions with low moisture, such as incineration of solid waste and the nitrogen fertilizer industry. As shown in Figure 1, the NH_3_ treatment capacity for PA0 is about three times that of PC0. Moreover, it could be that water in the acidic porous polymer is less easy to remove in the treatment process, as opposed to the activated carbon; the adsorption capacity decreased about 15% for PA14 from PA0, in contrast to about 70% from PC0 for PC8.

### 3.2. Structure of H_3_PO_4_/C and H_3_PO_4_/A Characterized by X-ray Diffraction (XRPD)

**Figure 2:**
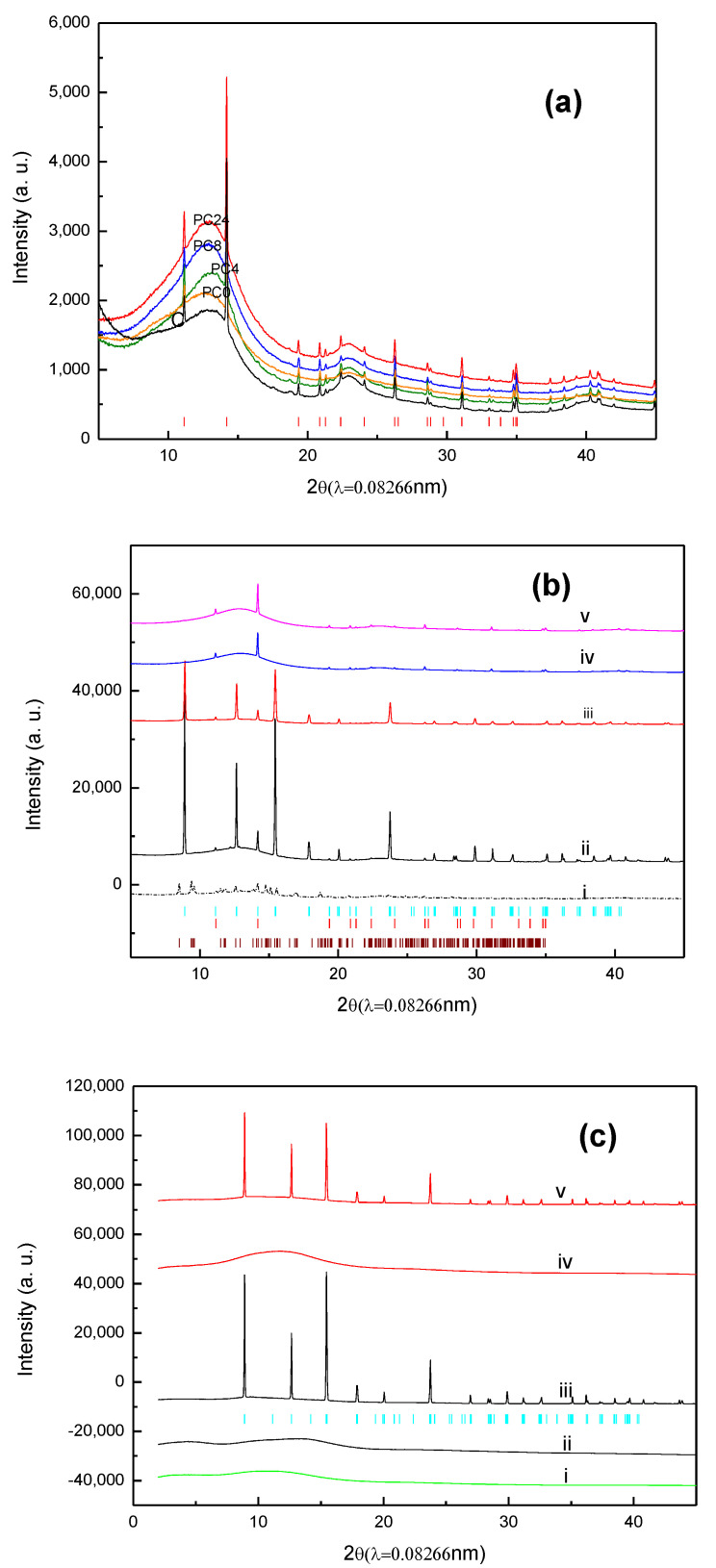
(**a**). Powder X-ray spectra of activated carbon (black), PC0 (orange), PC4 (green), PC8 (blue), and PC24 (red), red bar denotes SiO_2_; (**b**). Powder X-ray spectra of H_3_PO_4_/C after breakthrough test: (i) PC0_wet_ (dash-dot line), (ii) PC0_dry_, (iii) PC4_dry_, (iv) PC8_dry_, (v) PC24_dry_, blue bar denotes diffraction position of MAP, red bar denotes SiO_2_, and brown bar denotes DAP; subscript wet denotes testing by moisture containing waste gas, dry denotes by dry waste gas; (**c**). Powder X-ray spectra of H_3_PO_4_/A before and after breakthrough test: (i) Amberlyst 35; (ii) PA0; (iii) PA0_dry_; (iv) PA14; (v) PA14_dry_. blue bar denotes the diffraction position of MAP.

XRPD patterns characterizing the species on the fresh PC, used PC, combined fresh and used PA were shown in Figure 2a–c, respectively. The crystalline substances of the samples having long-range order lead to the appearance of Bragg peaks in X-ray diffraction. These substances can be identified by matching the XRPD pattern with reference patterns of pure substances. For amorphous solids and liquids, their structures lack periodicity. So, the diffraction from them does not show sharp Bragg peaks. Instead, X-rays will be scattered in many directions of these substances from tightly packed atoms leading to one or two broad maxima of the XRPD pattern [29].

Both broad backgrounds produced by carbon and sharp Bragg peaks by crystalline phase of SiO_2_ were present for activated carbon (GAC830), the raw material of PCs (Figure 2a). Comparing PCs with GAC850, the Bragg peaks of SiO_2_ remained intact, whereas the background intensity increased with drying time and the peak shift from 13.2 to 12.7 degree of 2θ. Since the H_3_PO_4_ solution for impregnation was prepared by dissolving P_2_O_5_ in water, the additional noncrystalline intensity contributions [24,29] could be due to the formation of amorphous P_2_O_5_ and/or H_3_PO_4_ in drying. 

After the aging test, XRD peaks characterizing DAP [(NH_4_)_2_HPO_4_] were observed for the moisture-containing waste gas, in contrast to MAP [NH_4_ H_2_PO_4_] for dry NH_3_ waste gas (Figure 2b). The intensity of the MAP peaks is decreased with the drying time; after 8 h of drying, almost no significant MAP characteristic peaks were observed. These results suggest: (1) NH_3_ in the dry gas stream can be removed by PC with sufficient water containing via both adsorption and neutralization reaction, (2) NH_3_ is removed by adsorption for PC without sufficient water; and (3) when NH_3_ in the wet gas stream, MAP is converted to DAP during the tests.

**Figure 3:**
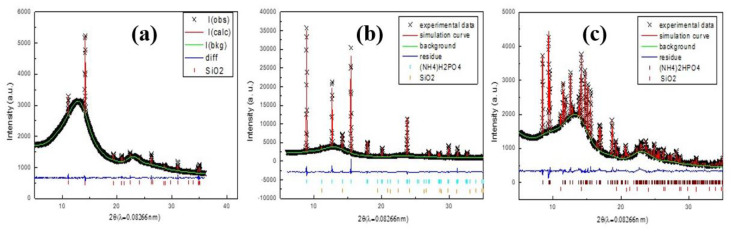
The Rietveld refinement of PC24_dry_ (**a**), PC0_dry_ (**b**) and PC0_wet_ (**c**). (+) denote experimental data, solid red line denotes simulation curve, green line denotes baseline, blue line denotes residue, blue bar denotes diffraction position of ADP, red bar denotes SiO_2_, and brown bar denotes MDP.

The Rietveld refinement results confirm the formation of MAP for PC0, PC4, AC0, and AC8 in the dry gas stream and DAP on PC0 in the wet gas stream. Besides confirming the phase assignment of XRPD patterns, crystallinity, unit cell parameters, and the presence of preferred orientation can be further quantified using Rietveld refinement [24]. The final Rietveld refine results are shown in Figure 3, and the refined crystal parameters were summarized in Table 1. 

No preferred orientation was detected in the XRPD patterns, suggesting the formation of a spherical crystal. The crystalline grain sizes were obtained from the commonly used Scherrer’s equation, *t* = *kλ*/Bcosθ, with the crystal grain size *t*, shape correction constant *k* = 0.95 for spherical particle, and B = FWHM of the related Bragg peaks [26]. The estimated crystal size is also shown in Table 1. 

The relative MAP or DAP crystal formed on PCx, ACx, to that formed on PC0 calculated from Bragg peaks and NH_3_ removal capacity are shown in Table 2. 

### 3.3. Mechanism of NH_3_ Removal Using PC and PA

**Figure 4:**
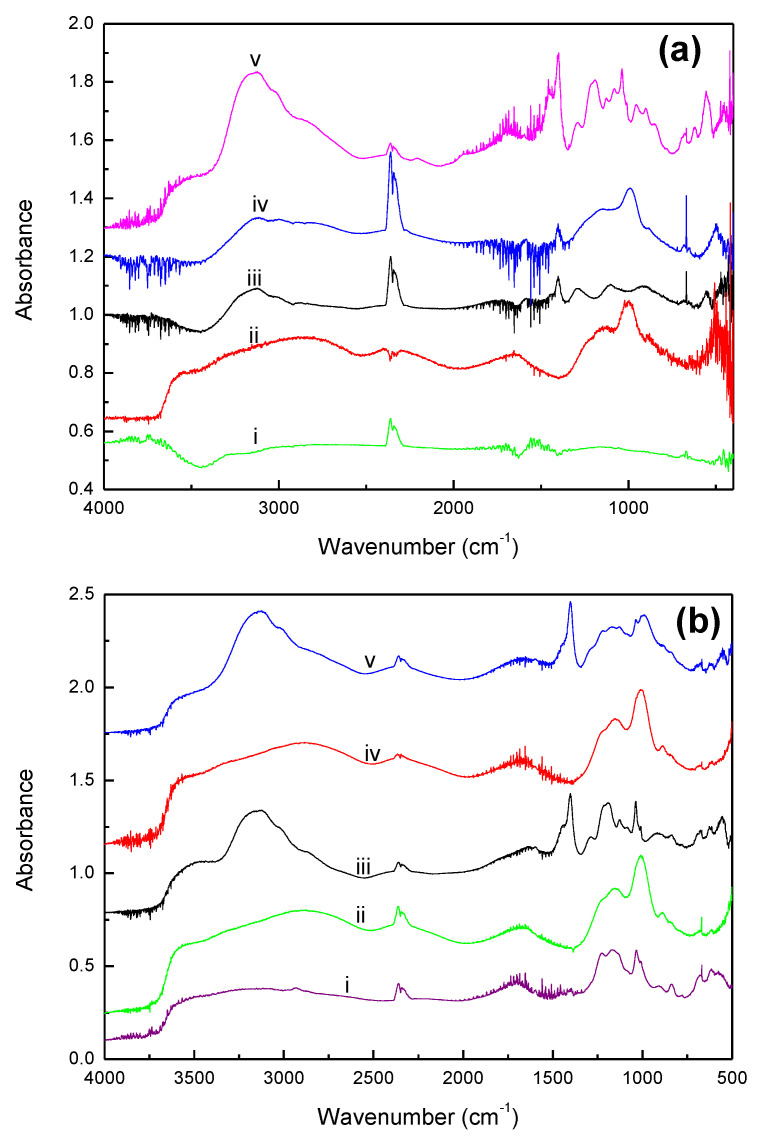
(**a**). In situ FT-IR spectra of (i) activated carbon, (ii) PC0, (iii) PC0 after flowing NH_3_ in dry air, (iv) PC8 after flowing NH_3_ in dry air, and (v) PC0 after flowing NH_3_ in wet air; (**b**). In situ FT-IR spectra of (i) Amberlyst 35, (ii) PA0, (iii) PA0 after flowing NH_3_ in dry air, (iv) PA14, and (v) PA14 after flowing NH_3_ in dry air.

The IR spectra characterizing GAC830, fresh PC0, PC0 after in situ dry (PC0_id_) and wet NH_3_ air testing (PC0_iw_), and PC8 after dry testing (PC8_id_) are shown in Figure 4a.

The characteristics of O-H stretching (ν_OH_) and bending vibration (δ_OH_) bands at 3600 and 1665 cm^−1^ were observed for fresh PC0 and PC0_iw_. Compared with liquid water (ν_OH_ = 3400, δ_OH_ = 1640 cm^−1^) [30], the blue shift of O-H bands to higher frequency suggests that the water molecules are chemisorbed on the sorbents, dissociated to proton and hydroxyl group, and then bonded to the acid sites of the sorbents [30]: the phosphate of H_3_PO_4_. The characteristic peaks of PO_4_ for PC0 and PC8_id_ showed at about 1200 (ν_asPO_), 850 (ν_sPO_), and 1010 (ν_PO-H_) cm^−1^, while the OH group bonded to PO_4_ showed at 3150 cm^−1^ [31,32]. For AC8_id_, the disappearance of 3600 and 1665 cm^−1^ peaks suggests water evaporation during the tests. The appearance of the NH_4_^+^ deformation band at 1400 cm^−1^ [30,33] concomitant to water desorption could be due to the onset of MAP formation; MAP could be formed by NH_3_ adsorption followed by surface reaction.

It could be because of more water on PC0_id_ than on PC8_id_, crystal MAP formed on PC0. MAP formation is evidenced by the absorption band appearing at 1290 cm^−1^, which is a combination of the asymmetric _S_tretching vibration of PO_4_ with lattice [33]. The NH_4_ of MAP is characterized by the peak appearing at 1400 cm^−1^ (δ_NH_) and the peaks at 3140, 3000, and 2850 cm^−1^ (ν_NH_). The peaks at 1100 and 900 cm^−1^ are attributed to P-O vibrations and P-O-H (ν_PO-H_) of PO_4_ [33]. 

When wet air was introduced, the appearance of bands at 3600 and 1670 cm^−1^ indicate the association of water with PC0 [30]. Moreover, the appearance of peaks at 1450 (δ_NH_), 1190 (ν_PO-H_), and 1060 cm^−1^ (ν_PO-H_) suggest the presence of NH_4_^+^, H_2_PO_4_^−^, and HPO_4_^2−^ [30,34,35]. 

Based on XRPD and FT-IR results, the chemistry involved in the NH_3_ removal process is formulated as the following equations:

P_2_O_5(s)_ + 3H_2_O_(l)_ ⇌ 2H_3_PO_4(aq)_ (1)

H_3_PO_4(aq)_ ⇌ H_3_PO_4_⋯H_2_O_(s,gel)_ + P_2_O_5(s)_ + H_2_O_(g)_ (2)

H_3_PO_4_⋯H_2_O_(s,gel)_ + NH_3(g)_ → (NH_4_)H_2_PO_4(s,gel)_ + H_2_O_(g)_ (3)

(NH_4_)H_2_PO_4(s,gel)_ + NH_4_OH_(aq)_ → (NH_4_)_2_HPO_4(s)_ + 2H_2_O_(g)_ (4)

The subscript s, l, g, gel, and aq are noted in the equations above as solid, liquid, gas, gelation, and aqueous phase. 

In the first step of PC preparation (Equation (1)), the viscosity of 7M (37 %) H_3_PO_4(aq)_ for impregnation preparation of H_3_PO_4_/C is 7.8 cp (10^−3^ Kg/m.s). During the drying step (Equation (2)), H_3_PO_4(aq)_ viscosity increases concomitantly with the evaporation of water molecules on the adsorbents, leading to gel-like substances. For PC0, H_3_PO_4(aq)_ concentration in the sorbents is about 85%. At room temperature, the measured viscosity of 85% H_3_PO_4(aq)_ is about 97 cp. After drying by flowing air at 120 °C for more than 4 h, some of the H_3_PO_4_ were even converted to amorphous P_2_O_5_. 

During dry air testing, NH_3(g)_ is transported from the waste gas stream to the external surface of the PC and reacted with H_3_PO_4_⋯H_2_O to form (NH_4_)H_2_PO_4(s)_ (Equation (3)). When sufficient water molecules were present on PC, such as PC0, (NH_4_)H_2_PO_4(s)_ were crystallized to form crystals. These crystals of size about 30 nm (Table 2) could block mesopores of PC for the further reaction of H_3_PO_4_⋯H_2_O with NH_3_. On the other hand, when insufficient water molecules on PC, such as PC08, amorphous P_2_O_5_ formed in the drying step block the diffusion of NH_3_ inside PC pores. Hence, only an external pellet surface can adsorb NH_3_ for reaction.

In wet air testing, the reaction mainly occurs in the aqueous phase. The water vapor scrubbed from a water reservoir is condensed on the reactive adsorption bed. The aqueous NH_4_OH then react with MAP to form DAP (Equation (4)).

**Figure 5:**
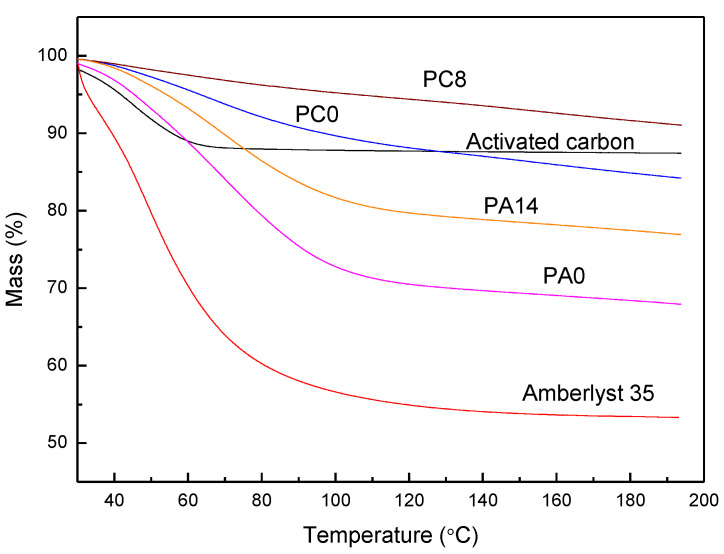
TGA profile of activated carbon (black), Amberlyst 35 (red), PA0 (pink), PA14 (orange), PC0 (blue), and PC8 (brown).

Using active carbon, zeolite as adsorbents for NH_3_ removal, moisture competes with NH_3_ for adsorption, leading to a reduction of adsorption. However, when H_3_PO_4_ impregnated adsorbents (PIA) are used, moisture is necessary to increase NH_3_ removal capacity. In removing ammonia from waste gas with low moisture by PIA, water evaporation from the support surface is inevitable. To keep the NH_3_ removal efficacy, the IPA needs support having high *moisture*-retaining capability. Amberlyst 35 was used as the support because it has 52 to 57% retention capacity due to its surface sulfuric acid functional groups. As shown in Figure 5, characterized by TGA, the water contained in PC0, PC8, PA0, PA14 is 16.0, 9.0, 32.1, and 23.4%, respectively (Table 2). For PC8, the water content is lower than the stoichiometric calculation of % water in H_3_PO_4_/C (about 10% water). The results indicate that some H_3_PO_4_ were dehydrated to P_2_O_5_, consistent with the inference from XRPD patterns. 

The characteristics of the water species on PA0 and PA14 are similar to PC0; peaks at 3600 and 1665 cm^−1^ suggest water associated with H_3_PO_4_ (H_3_PO_4_⋯H_2_O). As expected, due to higher water retention capacity, MAP is formed by neutralization in the aqueous phase. Besides crystal MAP, the presence of aqueous MAP is evidenced by the presence IR absorption band for NH_4_^+^ at 1445 and 1033 cm^−1^, and H_2_PO_4_^−^ at 1190 cm^−1^. Moreover, the presence of IR peaks at 3600 and 1650 cm^−1^ for PA0 and PA14 (Figure 4b) suggests water was unlikely to be desorbed in the NH_3_ capture process by using Amberlyst 35 support. 

**Figure 6:**
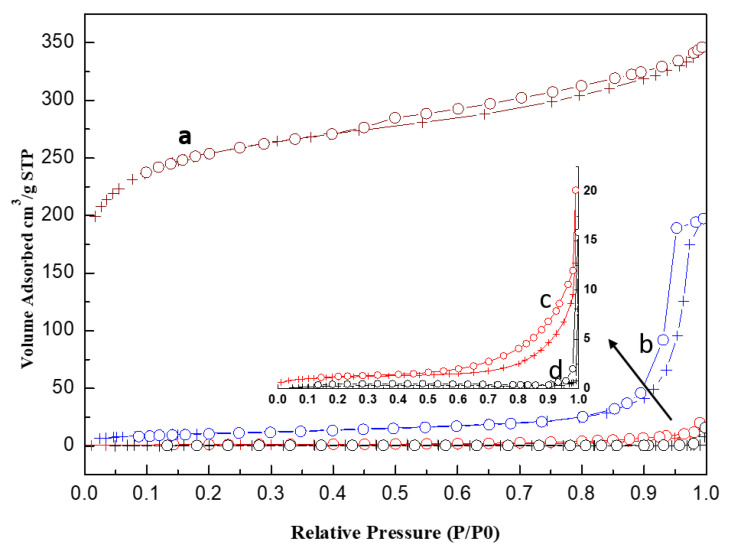
Nitrogen adsorption isotherm at 77K for (**a**) PC0, (**b**) AC0, (**c**) used PC0, and (**d**) used PC0.

As shown in Figure 6, the N_2_ isotherm for activated carbon (GAC830) is concave to x (P/P0) axis in the range of P/P0 = 0.0 to 0.40 and the isotherm is then convex to the x-axis. The sample is assigned as IV(a) type isotherm. For acidic porous polymer (Amberlyst 35), the N_2_ isotherm only has a slight convex to the x-axis in the range of P/P0 = 0.0 to 0.20. The sample is assigned as mixed IV(a) and V type [36,37].

The shape of the hysteresis loop is related to the shape of the pores. With the loop of upward curvature at a relative pressure (P/P0) between 0.8 and 0.98 (H_1_ hysteresis loop), the pore of the acidic porous polymer is of cylindrical type. In contrast, with a slow jump in the H_4_ hysteresis loop at 0.45–0.95 P/P0 region, the activated carbon is a mesoporous material with slit-type porosity [37].

Compared with acidic porous polymer, the activated carbon comprises a much smaller pore diameter and larger surface area. If the NH_3_ removal process is dominated by adsorption, activated carbon is expected to have a higher NH_3_ removal capacity. However, the reverse was observed for the experimental data, confirming the neutralization dominates the process.

**Figure 7:**
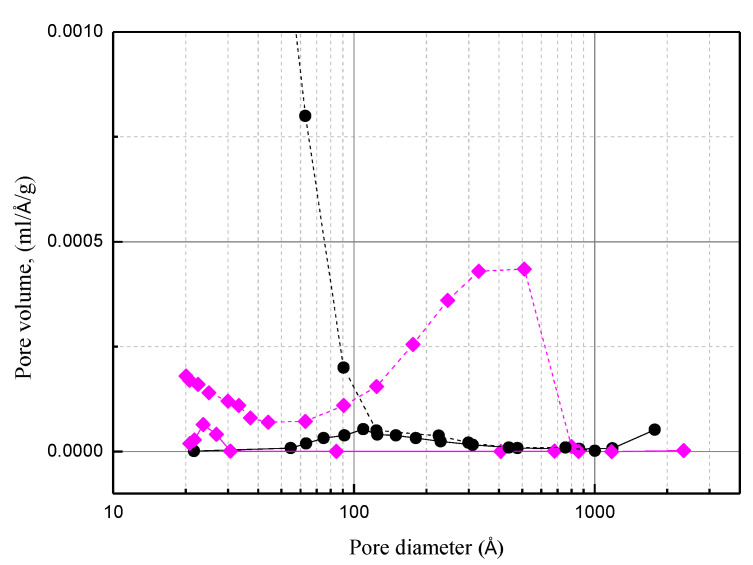
Comparison of pore size distribution for the activated carbon (dash line ●), Amberlyst 35 (dash line ♦), used PC0 (solid line ●), used PA0 (solid line ♦).

After impregnating H_3_PO_4_ and breakthrough testing, the hysteresis loop changed to H_2_ type (PC0) (Figure 7). Moreover, the comparison of the pore size distribution for the used PC0 with that for carbon support indicates that phosphorus species were not fully accommodated in the mesopores (>100 Å). Because of the weak affinity of water to the carbon support, water in mesopores is easy to evaporate and diffuse out. Due to higher diffusion resistance and the adsorption on the pore wall, water in the micropores is harder to diffuse out [26]. In contrast, by using acidic porous polymer, because water molecules are strongly bonded by the sulfonic acid group, which promotes the reaction of NH_3_ with H_3_PO_4_, almost all the mesopores of acidic porous polymer were accommodated by the phosphorus species.

In our previous works, H_2_SO_4_/C was prepared by impregnating the activated carbon with a 7.9 M sulfuric acid solution. The NH_3_ removal capacity of H_2_SO_4_/C sorbent for wet gas treatment is about 70 g NH_3_/Kg [10], which is slightly higher than that of H_3_PO_4_/C (PC0) for wet gas treatment but lower than H_3_PO_4_/A (PA0) for dry gas (Table 2). The comparison demonstrates the merits of using a functionalized porous polymer to prepare sorbents to capture acid or base in the waste gas stream.

The used H_2_SO_4_/C sorbent can be reused by washing away the (NH_4_)_2_SO_4_ formed on the activated carbon in the NH_3_ capture process, followed by drying and resoaking sulfuric solution. In the washing step, low-pressure steam or hot water can be used to dissolve (NH_4_)_2_SO_4_ crystals [10]. Since the solubility of MDP in water is close to that of (NH_4_)_2_SO_4_ [38,39], similar procedures can be used to rejuvenate used H_3_PO_4_/C and H_3_PO_4_/A. For H_2_SO_4_/C, the performance of the rejuvenated one is close to the fresh sorbents [10]. Since H_3_PO_4_ tend to be less corrosive than H_2_SO_4_, using H_3_PO_4_, the damage of the support structure in the NH_3_ capture process will be less severe. Therefore, the stability maintenance for H_3_PO_4_/C is expected to be better than that of H_2_SO_4_/C.

## 4. Conclusions

Capturing NH_3_ in dry air waste gas and water containing the sorbents shows a significant role in process efficacy. Water facilitates ammonium ions formation. The ions are either bonded to carbon or acidic polymer or react with dihydrogen phosphate ions to form MDP. Due to water evaporation in the process, experimental results indicated that no MAP crystal was observed for water containing H_3_PO_4_/C is less than 9% (PC8). MAP crystal was formed for water containing H_3_PO_4_/C to reach 16% (PC0), whereas the stoichiometric calculation indicated that essentially not all of the H_3_PO_4_ on the activated carbon were reacted with NH_3_. The results combined with the pore size distribution suggested that at a high concentration of H_3_PO_4_, the quick evaporation of the water deposited on the mesopores (>100 Å) hinders ammonium ions’ formation. When the process occurs in wet gas, moisture condensation may partially compensate for water evaporation. Hence, the NH_3_ removal capacity of PC0 for wet gas treatment is more than two times that for dry gas. The experimental results thus suggest H_3_PO_4_/C is more suitable for moisture-containing waste gas. However, the drawbacks of H_3_PO_4_/C for capturing NH_3_ in a dry gas stream can be remedied by using acidic porous polymer supports. Compared with H_3_PO_4_/C, water is more likely to be retained on H_3_PO_4_/A due to the presence of sulfonic functional groups and cylindrical type pore structure. Experimental results indicated that the adsorption capacity for PA0 used in dry gas is more than three times that of PC0.

## Figures and Tables

**Table 1:** The results of crystal parameters by the Rietveld refinement.

Sample Name	PC24_dry_	PC0_dry_	PC0_wet_
Space group: SiO_2_	P3221(trigonal)	P3221 (trigonal)	P3221 (trigonal)
H_2_(NH_4_)PO_4_	-		-
H(NH_4_)_2_PO_4_	-	-	P21/C (monoclinic)
Lattice parameters:			
H_2_(NH_4_)PO_4_(Å)	-	a = 7.477, c = 7.528	-
H(NH_4_)_2_PO_4_(Å)	-	-	a = 10.959, b = 6.651, c = 7.967
Lattice parameters:	a = 4.918	a = 4.899	a = 4.891
SiO_2_(Å)	c = 5.409	c = 5.392	c = 5.380
wRp	0.0094	0.038	0.020
Rp	0.0058	0.025	0.014
X^2^	0.082	0.106	0.136
Grain size (nm)			
SiO_2_	54	53	42
H_2_(NH_4_)PO_4_	-	32	-
H(NH_4_)_2_PO_4_	-	-	39

**Table 2:** Effects of water content on NH_3_ removal capacities and the ratio of crystal formed in sorbent x to that in PC0; removal capacities were estimated from breakthrough curves and Crystal(x)/Crystal (PC0) from Rietveld refinement results.

Sorbent x	Water % Mass	P_2_O_5_ % Mass	g (Mole) NH_3_/ Kg Sorbent	Crystal(x)/ Crytstal(PC0)
PC0	16.0	26.3	28(1.64)	1
PC0 (wet)	16.0	26.3	66(3.88)	0.145
PC4	9.0	30.9	11(0.64)	0.284
PA0	32.1	18.0	94(5.53)	1.574
PA14	23.4	23.9	78(4.56)	1.198

## Data Availability

Not applicable.
